# Subchronic Treatment with CBZ Transiently Attenuates Its Anticonvulsant Activity in the Maximal Electroshock-Induced Seizure Test in Mice

**DOI:** 10.3390/ijms252413563

**Published:** 2024-12-18

**Authors:** Monika Banach, Kinga K. Borowicz

**Affiliations:** Independent Experimental Neuropathophysiology Unit, Chair and Department of Toxicology, Medical University of Lublin, Jaczewskiego 8b, PL-20-090 Lublin, Poland; monikabanach@umlub.pl

**Keywords:** carbamazepine, antiseizure drugs, chronic treatment, epilepsy, maximal electroshock-induced seizures

## Abstract

The objective of this study is to evaluate the anticonvulsant efficacy of carbamazepine (CBZ) following acute and chronic administration across four treatment protocols in a murine model of maximal electroshock-induced seizures. A single dose of the drug was utilized as a control. The neurotoxic effects were evaluated in the chimney test and the passive avoidance task. Furthermore, plasma and brain concentrations of CBZ were quantified across all treatment protocols. The subchronic administration of CBZ (7 × 2 protocol) resulted in an attenuation of its antielectroshock effect. In the three remaining treatment regimens (7 × 1, 14 × 1, and 14 × 2) the median effective doses of CBZ were comparable to the control. Neither acute nor chronic treatment with CBZ resulted in a discernible impact on motor coordination or long-term memory. The plasma and brain concentrations of CBZ were significantly lower in most chronic protocols when compared to a single-dose application. This may explain the transient attenuation of CBZ effectiveness in the 7 × 2 protocol, but not the return to the previous level. The anticonvulsant and neurotoxic profiles of CBZ did not differ after single and chronic administration. Therefore, experimental chronic studies with CBZ are not prerequisites for concluding and possibly translating results to clinical conditions.

## 1. Introduction

Despite the introduction of second- and third-generation antiseizure drugs (ASDs) and the use of these drugs in combination therapy, approximately 30% of epilepsy cases remain refractory to pharmacological treatment. Regardless of considerable effort, the underlying causes of drug resistance remain unknown. Consequently, the search for new ASDs and their combinations that result in synergistic (or at least additive) interactions in terms of therapeutic effects, and antagonistic (or additive) interactions in terms of adverse effects, is ongoing. Such studies are conducted in experimental settings. However, the majority of them are conducted using a single drug model. Concurrently, the treatment of epilepsy is a prolonged process, typically lasting at least two years and, in some cases, a lifetime. In order to more accurately reflect real-world clinical conditions, ASDs should be administered in a chronic model, where pharmacokinetic and pharmacodynamic processes can develop. This may subsequently alter the efficacy of a given drug, the intensity of induced side effects, and the effect of interactions with other drugs. The mechanisms of these alterations can be further investigated in more advanced studies.

It is, therefore, recommended that preclinical studies be conducted using a chronic drug administration model, as this will facilitate the extrapolation of results to clinical conditions with greater reliability. This approach may also help to reduce the discrepancy between the results of preclinical and clinical studies.

The present study was conducted using the maximal electroshock (MES) test in mice.

This test, developed over 70 years ago, is arguably the most rigorously validated preclinical test for predicting drugs effective against generalized tonic–clonic seizures. The test is well-standardized, straightforward to perform, and necessitates low investment in equipment and technical competencies. The high correlation between the efficacy of ASDs in the MES test in mice and in epilepsy patients, coupled with the aforementioned arguments, are the primary reasons for the continued prevalence of this model in experimental studies. The MES test is predominantly intended, but not limited, to assess the efficacy of drugs targeting Na+ channels, including carbamazepine. Conversely, the majority of established and novel ASDs have demonstrated effectiveness in the MES test, even though they interact with other pharmacological targets [1].

Traditionally, first-generation ASDs were the drugs of first choice in new-onset epilepsy treatment. One of them was carbamazepine (CBZ), an iminostilbene derivative that was initially developed as an antipsychotic medication by Walter Schindler in 1953. Subsequently, it proved to be an efficacious treatment for seizures [2,3]. For a considerable period of time, CBZ was the preferred initial treatment option for patients experiencing focal seizures. In 2016, the medication was administered to 38 million patient-years, and it remains one of the most commonly prescribed ASDs [4]. In accordance with the recommendations set forth by the National Institute for Health and Care Excellence (NICE) in 2022, CBZ is currently recommended as a second-line monotherapy or add-on treatment option in the management of focal seizures. It is noteworthy that CBZ has been observed to potentially exacerbate absence, myoclonic, tonic, or atonic seizures [5]. Additionally, CBZ has been recommended for the treatment of trigeminal neuralgia and bipolar disorders [6,7]. In animal models, CBZ has been shown to possess anticonvulsant properties in both maximal electroshock and amygdala kindling seizure models in rodents [8].

Recent reports indicate a notable decline in first-generation ASDs prescriptions and a growing inclination toward newer antiepileptics [9]. This process also affects CBZ, despite its wide availability, comparable efficacy to newer medications, lower price [10], and positive psychotropic activity, which is especially desirable in epileptic patients [11]. However, the declining application of the medication is attributed to its unfavorable safety profile and enzyme induction potential, which may lead to interactions with other drugs that are less favorable [9].

The adverse effects associated with CBZ treatment encompass an array of manifestations, including elevated aminotransferase level, temporal leucopenia, dose-dependent fatigue, diplopia, vertigo, emesis, vomiting, and hyponatremia. The most serious medical complications are as follows: Stevens–Johnson syndrome, aplastic anemia, and carbamazepine hypersensitivity syndrome, which presents with fever, lymphadenopathy, and toxic liver injury [12,13].

In the present study, we sought to evaluate the anticonvulsant efficacy of CBZ following acute, subacute, and chronic regimens (implemented in four distinct protocols) in a murine model of maximal electroshock-induced seizures. Plasma and brain concentrations of CBZ following single and repeated administration in the same treatment regimens were determined. Furthermore, motor performance and long-term memory were assessed using the chimney test and passive avoidance task, respectively.

## 2. Results

### 2.1. Maximal Electroshock

The antielectroshock activity of CBZ varied depending on the chronic treatment regimen. Each chronic protocol was followed by a new control (single dose of the drug) to exclude seasonal influences on the anticonvulsant activity of the tested AED [14]. The ED_50_ value of CBZ applied in the 7 × 2 protocol was significantly higher than the control value (acute protocol): 15.7 ± 1.2 mg/kg vs. 10.5 ± 0.9 mg/kg [t(54) = 2.6; *p* = 0.012]. No significant differences were observed in the three other treatment protocols.

The detailed results of the MES test are as follows:

1. A single injection was administered every 24 h for 7 days (7 × 1; subchronic treatment).
  Control: 11.5 [9.5–13.7] mg/kg
  7 × 1: 13.8 [12.1–15.7] mg/kg

2. Two injections were administered every 12 h for 7 days (7 × 2 subchronic treatment).
  Control: 10.5 [8.8–12.5] mg/kg
  7 × 2: 15.7 [13.5–18.3] mg/kg (*p* < 0.05)

3. A single injection was administered every 24 h for 14 days (14 × 1 chronic treatment).
  Control: 13.5 [12.2–15.1] mg/kg
  14 × 1: 14.3 [12.7–16.1] mg/kg

4. Two injections daily were administered every 12 h for 14 days (14 × 2 chronic treatment).
  Control: 14.2 [12.8–15.9] mg/kg
  14 × 2: 16.8 [14.9–18.9] mg/kg

**Figure 1:**
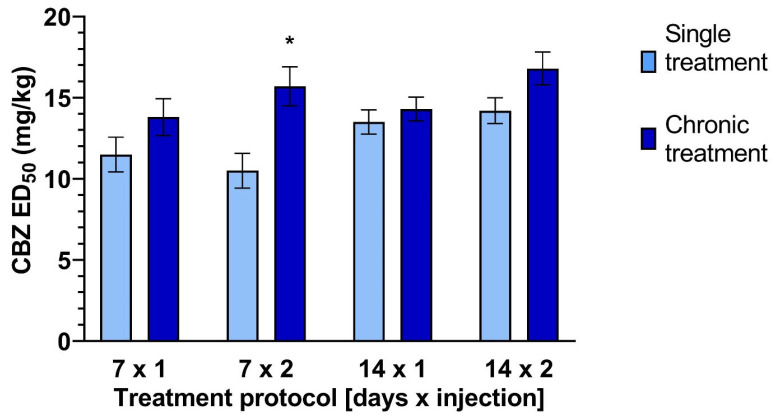
Effects of acute and chronic (four protocols) treatments with carbamazepine (CBZ) on anticonvulsant activity against maximal electroshock-induced seizures in mice. Data are presented as median effective doses (ED_50_ with SEM values), which protected 50% of the animals from seizures. CBZ was administered acutely or chronically (in four protocols) 30 min prior to the testing; treatment protocols: 7 × 1, 1 injection daily for 7 days; 7 × 2, 2 injections daily for 7 days; 14 × 1, 1 injection daily for 14 days; 14 × 2, 2 injections daily for 14 days; * *p* < 0.05 vs. control (single administration of CBZ).

The effects of CBZ administration on maximal electroshock-induced seizures are presented in Figure 1.

### 2.2. Chimney Test

CBZ administered acutely or chronically (14 × 2) at doses of 14.2 mg/kg (ED_50_1) and 16.8 mg/kg (ED_50_2) did not affect motor coordination in the chimney test in mice (Table 1).

### 2.3. Step-Through Passive Avoidance Task

Neither acute nor chronic CBZ treatment at either dose of ED_50_1 and ED_50_2 affected long-term memory in mice (Table 2).

### 2.4. Plasma and Brain Concentrations of CBZ

**Figure 2:**
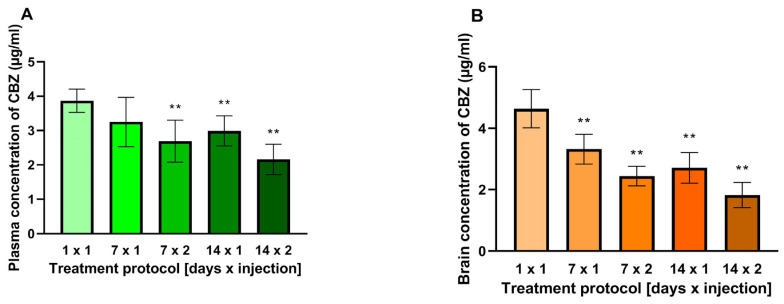
Plasma (**A**) and brain (**B**) concentrations of carbamazepine (CBZ) after acute and chronic treatments. Results are presented as mean ± SD of at least eight determinations. Statistical analysis of the plasma and the brain concentrations of CBZ was performed using the one-way analysis of variance (ANOVA) followed by post hoc Dunnett’s test. A single dose administration of CBZ served as a control. CBZ–carbamazepine; treatment protocols: 7 × 1, 1 injection daily for 7 days; 7 × 2, 2 injections daily for 7 days; 14 × 1, 1 injection daily for 14 days; 14 × 2, 2 injections daily for 14 days; ** *p* < 0.01 vs. control (single administration of CBZ).

The plasma concentration of CBZ measured in the subchronic protocol of 7 × 1 was not significantly different from the control (1 × 1). However, the plasma concentration of CBZ was significantly lower than the control [F(4.35) = 11.48; *p* < 0.0001] in the remaining chronic protocols (7 × 2, 14 × 1 and 14 × 2). Brain concentrations of CBZ were significantly lower in all four chronic protocols when compared to the control [F(4.35) = 40.36; *p* < 0.0001] (Figure 2).

## 3. Discussion

The efficacy and safety of CBZ in humans were evaluated in 150 trials, which can be found on the clinicaltrials.gov website [15]. CBZ has often served as the standard comparator for newer ASDs, confirming its established position as a potent anticonvulsant. An important feature of CBZ is its enzyme induction potential, which may influence its antiseizure activity and safety profile, as well as the risk of drug–drug interactions [10]. This led us to investigate this drug in our work.

In the preclinical phase of drug evaluation, active molecules are usually administered as a single injection. Based on our previous experimental data, we are aware that anticonvulsant potency and side-effect profiles can change depending on the duration of therapy [16,17]. Here, we present the results of our following scientific project dealing with chronic CBZ administration in mice. The antiseizure effect of prolonged treatment with CBZ was evaluated in the MES test and compared with control groups of animals, which received only one dose of the drug. In one case, a significant difference was found between the calculated ED_50_s in the tested groups and the controls: 7 × 2 vs. 1 × 1. Subchronic (7 × 2) application of the ASD resulted in a significantly higher ED_50_ value than a single dose of the drug, indicating a decrease in its antiseizure potency.

Confronting the obtained results with literature data proved to be extremely difficult because observations on the effects of chronic administration of CBZ in animals are very scarce. In rats, CBZ administered at a dose of 100 mg/kg (t.i.d. orally) for 5 days significantly reduced the frequency of kainic acid-induced seizures. A maximal anticonvulsant effect was achieved on the second day of treatment and was maintained even after discontinuation of the drug [18]. A lower dose of CBZ 40 mg/kg (t.i.d. i.p.) administered for two weeks successfully reduced spontaneous epileptic seizures in the rat pilocarpine model [19]. Chronic treatment with CBZ (15 mg/kg per os for up to 18 days), but not repeated intraperitoneal injections, inhibited seizure development induced by cocaine or lidocaine in rats. It did not affect fully developed seizures in rats [20]. Similarly, chronic treatment with CBZ was observed to inhibit cocaine-induced seizures in genetically modified mice (BALB/cByJ, C57B1/6J, SJL/J), with the maximal effect occurring after 4–7 days. The protective effect was sustained for a period of 5 days following the cessation of treatment [21]. In the amygdala-kindling model in rats, chronic administration of CBZ (30 mg/kg t.i.d. i.p. for two weeks) resulted in the development of tolerance to the anticonvulsant effect as demonstrated by Hönack and Löscher [22]. This phenomenon has been corroborated by other researchers in the context of the administration of this AED prior to, but not subsequent to, the electrical stimulation of rats [23,24,25,26]. In light of the aforementioned studies, it can be posited that chronic treatment with CBZ results in potentiation or stabilization of its anticonvulsant effect against chemically-induced seizures. One exception was the amygdala-kindling model in rats which, similar to maximal electroshock, is electrically induced. In this model, a decrease in the antiseizure effect was observed. However, no available study demonstrated transient attenuation of the antiseizure efficacy of CBZ during chronic administration. The inference based on the above data is limited by the fact that the majority of the above studies were conducted on rats.

In a recent article, we demonstrated that chronic treatment with oxcarbazepine, a 10-keto analog of CBZ, resulted in a reduction of its anticonvulsant activity in the MES test in mice in the two-week protocols. The ED_50_ values of OXC, as indicated in the 14 × 2 and 14 × 1 protocols, were considerably higher than those of the corresponding dose in the control groups (1 × 1). The observed alterations were dependent rather on pharmacodynamic factors, as chronic treatment with oxcarbazepine did not result in any change to its plasma or brain levels. The most significant molecular target for oxcarbazepine is the voltage-gated sodium channel. Similarly to the presented results of this study, adaptive alterations affecting ionic channels may elucidate the phenomenon of reduced therapeutic effect following chronic oxcarbazepine administration [17].

Conversely, the assessment of the clinical efficacy of ASDs may be impeded by a number of conflicting factors, including inappropriate drug administration, noncompliance, and the progression of the underlying disease. For example, after the exclusion of potential confounding factors, an unanticipated worsening of epilepsy was observed in 5% of patients [27]. In a study of Canadian epileptic children, a decrease in the antiseizure action of CBZ was observed in 4% of patients within 12 months [28]. In adults with focal seizures treated with CBZ, 63% of patients exhibited seizure remission three years after the initial target dose was administered. The treatment was maintained for a period of 3 years in 36.1% of patients [29]. In a separate investigation, 85.55% of patients with focal epilepsy exhibited complete seizure control over the course of a 12-month period. Nevertheless, over 23% of patients were compelled to discontinue CBZ due to the unfavorable consequences of the therapeutic regimen [30]. In a cohort of healthy volunteers, chronic administration of CBZ (100–400 mg b.i.d. for 5 weeks) resulted in an elevation of the cortical motor threshold, as evaluated by transcranial magnetic stimulation. The outcome was found to be directly proportional to increasing plasma concentration of the drug. Following the cessation of pharmacotherapy, the initial effect was sustained for several days, despite the drug’s undetectable concentration [31]. More recent studies have demonstrated that second-generation ASDs are associated with higher rates of persistence and adherence compared to CBZ. A meta-analysis of 30 randomized clinical trials was conducted to investigate the seizure freedom rate and retention rate of CBZ. The 12-month seizure freedom rate was 48%, and the retention rate was 61%. The authors observed a high degree of variability in the results, which they attributed to the length of treatment and the use of blinding in the research [32]. Nevertheless, the aforementioned studies do not address the potential transient attenuation of CBZ effects during chronic therapy.

The subsequent phase of our investigation entailed an examination of the impact of prolonged CBZ administration on the neurotoxic undesired effects. Neither acute nor chronic treatment with CBZ resulted in any discernible impact on motor coordination as assessed in the chimney test in mice. Also, long-term memory, as evaluated in the passive avoidance task, was not impaired by CBZ in any treatment protocol. An attempt to calculate the toxic dose of CBZ in the chimney test was unsuccessful. In the acute protocol, the median toxic dose (TD_50_) value was determined to be 66.3 mg/kg. These findings align with those reported by other authors, such as the value of 53.3 mg/kg observed by Łuszczki et al. [33]. In the chronic protocol, despite dose escalation of up to 170 mg/kg, no potentiation of the neurotoxic effect was observed in the chimney test in mice. This may indicate the development of tolerance to the neurotoxic effects of CBZ. It is noteworthy that chronic administration of oxcarbazepine resulted in a notable reduction in neurotoxicity. The median toxic dose (TD_50_) obtained in the chimney test in mice in the most extensive chronic protocol was significantly higher than the control value (89.6 mg/kg vs. 66.4 mg/kg) [17].

The majority of experimental studies have demonstrated that there is no observable decline in memory during CBZ treatment. Conversely, the enhancement of cognitive functions was described in certain articles. These findings align with our results. Chronic administration of this ASD at a dose range of 5–80 mg/kg/day (i.p.) for 21 days did not affect learning and memory, as evaluated in the passive avoidance task and T-labyrinth test in rats, respectively. Furthermore, rats that received CBZ at doses of 20 and 40 mg/kg exhibited superior outcomes compared to the control group [34]. In a separate experiment, chronic administration of CBZ at a dose of 75 mg/kg every 6 h for 10 days (per os) did not affect rat behavior in the open field test [35]. Chronic administration of CBZ (40 mg/kg t.i.d. i.p. for 8 days) did not result in any locomotor disturbances or alterations in explorative behavior in a pilocarpine-induced seizure model in rats [36]. The repeated administration of CBZ at a dose of 5 mg/kg (i.p.) has been shown to prevent memory disruptions evoked by electroshock or ethanol in rodents [37]. Chronic treatment with CBZ resulted in enhanced outcomes in the active avoidance task in amygdala-kindled cats [38]. The chronic administration of CBZ (30 mg/kg b.i.d. per os for 7 and 14 days) did not result in any observable effects on motor coordination in the chimney test or memory in the Morris water maze test in rats [39]. In contrast, chronic administration of CBZ (30 mg/kg t.i.d. i.p. for 2 weeks) decreased the undesired effects observed in amygdala-kindled rats, namely ataxia, abdominal muscle relaxation, and body weight reduction [22]. A single study by Reeta et al. [40] demonstrated significant learning and memory impairments in rats following 21-day CBZ treatment (60 mg/kg, i.p.) in the passive avoidance task and elevated plus maze test. Moreover, intensification of oxidative stress, as indicated by elevated malondialdehyde and glutathione levels was observed. Noteworthy, curcumin, an antioxidant, prevented neurotoxic effects and reduced oxidative stress. The results of the passive avoidance task reported in this study are in contrast to ours. This may be due to different animal species and test parameters. Reeta et al. [40] used rats that were monitored for up to 600 s and treated with a current of 0.2 mA for 3 s. In contrast, mice in our study were exposed to an electric foot shock of 0.6 mA for 2 s.

In humans, CBZ is also thought to have a neutral or beneficial effect on cognitive function [41,42]. However, some studies reported psychomotor deterioration, attention deficits, and memory impairment [43,44]. As stated by Ries [45], in the majority of cases, CBZ does not impact memory in children. Other authors have, however, reported memory disturbances and learning difficulties in epileptic children treated with this drug [46,47]. In a retrospective study of patients with medial temporal lobe epilepsy treated with CBZ, Dusanter et al. [48] demonstrated deterioration in multiple cognitive domains. CBZ significantly worsened language functions, verbal episodic memory, and the delayed recall subtest of the Wechsler Memory Scale—Revised. However, the duration of treatment with this ASD in each patient was not mentioned, which appears to be an important limitation of this study.

In light of the aforementioned studies, it appears that chronic treatment with CBZ may confer benefits with respect to memory processes. The potential mechanisms contributing to this phenomenon may include elevated acetylcholine concentration and increased turnover of serotonin and dopamine in the hippocampus. In a study conducted by Sudha et al. [34], a reduction in acetylcholinesterase activity was observed in the hippocampus and piriform cortex. Additionally, therapeutic doses of CBZ were found to elevate serotonin levels by 36% and dopamine levels by 137% in the hippocampus.

When considering the results of the antielectroshock effects of CBZ, one potential explanation for the evolving profile of its activity during chronic administration is a pharmacokinetic mechanism. To substantiate this hypothesis, the plasma and brain concentrations of CBZ administered in both acute and chronic protocols were quantified. In the chronic protocols, except for the 7 × 1, the plasma concentration of CBZ was significantly lower than that observed in the control group (1 × 1). Concurrently, the concentration of CBZ in the mouse brain was markedly diminished in all chronic protocols when compared to the acute one. The diminished antielectroshock effect of the 7 × 2 protocol may be attributed to the reduced concentration of the drug, yet this does not explain the restoration of control values in the remaining protocols despite the sustained reduction in drug levels.

A review of the pharmacokinetic parameters of CBZ appears to be partially similar in rats and humans [35,49]. In humans, the maximal blood concentration of CBZ is reached after 4–8 h, while the stationary status can be attained after 2–4 days to 5 weeks of treatment [50,51,52]. The concentration of the drug in the cerebrospinal fluid is 17–31% of that in the plasma [52]. CBZ is metabolized by CYP 3A4, 2E1, and 2B6 cytochrome isoforms to 10,11-epoxide and 10,11-trans-dihydrodiol [53,54]. The primary metabolite, 10,11-epoxide, may attain elevated concentration in the brain (50% of blood concentration) and exhibit antiseizure properties [52,53,54,55,56]. CBZ metabolism exhibits a gradual and dose-dependent autoinduction [57,58]. As a consequence of this process, the half-life of CBZ in humans may undergo a significant alteration, ranging from 25–65 h at the outset of therapy to 12–17 h following stabilization of metabolism [53]. The therapeutic blood concentration in humans is 4–12 µg/mL, which can be reached after 3–5 weeks of treatment [59]. The serum concentration of carbamazepine epoxide is up to 2.3 µg/mL, which accounts for 19–57% of the parental drug [54]. In patients not previously exposed to CBZ, autoinduction typically begins in 3–4 days and can result in a 50% decrease in drug levels by the end of the 3-week treatment period [60].

The therapeutic plasma concentration of CBZ in rats is contingent upon the model of seizures, animal species, and the duration of treatment. In the MES and PTZ tests, the effective concentration (EC_50_) is 4–6 µg/mL (after a single dose), whereas in amygdala-kindled rats it is 16–22 µg/mL (after prolonged administration) [49]. The half-life of CBZ in the mouse plasma was assessed as 3.38 h, while that of the epoxide derivative occurred considerably longer (12.52 h). In the mouse brain, the corresponding values were 2.48 h and 7.78 h, respectively [61,62].

The measurement of blood concentrations of CBZ or its metabolites was seldom conducted in animal studies following the chronic administration. The concentration of CBZ and CBZ-10,11-epoxide in the brain and plasma decreased during a 9-day course of CBZ treatment in 3 strains of genetically modified mice [21]. In rats, the plasma concentration of CBZ was observed to be lower, whereas that of 10,11-epoxide was increased during chronic treatment lasting between 10 and 18 days [22,35]. It is noteworthy that in our study, the mean CBZ plasma concentration exhibited a significant decline from 3.8 µg/mL in control animals to 2.2 µg/mL in the most extensive protocol.

There is a method by which drug doses can be translated from animals to humans through the use of body surface area normalization [63]. If we assume that the ED_50_ value for CBZ in the acute protocol (14.2 mg/kg) represents a reasonable reference point for mice, then the calculated therapeutic human dose should be approximately 80.59 mg. After applying the correction for the oral route, this dose increases to 115.12 mg, which corresponds to the initiation dose of CBZ in epilepsy. Then the dose gradually increases to as much as 1200 mg/day. Given this, the levels of carbamazepine found in our study are not very different from the therapeutic levels of this drug in epilepsy patients. However, it is worth noting that the process of autoinduction is observed both in the studies presented here and in clinical settings. Hence, contrary to the common belief that the pharmacokinetics of drugs differ significantly between mice and humans, these differences appear to be much smaller in the case of carbamazepine.

In light of the aforementioned pharmacokinetic data, it is reasonable to conclude that the decrease in plasma and brain concentrations of CBZ during chronic administration is attributable to metabolic autoinduction. In the most comprehensive protocol, the plasma CBZ level decreased by 44%, while that measured in the brain decreased by 60%. It seems that the lowered antiseizure efficacy observed in the 7 × 2 protocol, was at least in part related to the significant reduction in plasma and brain concentrations of this ASD. However, this cannot explain the fact that in the 14 × 2 protocol, CBZ ED_50_ values returned to control values with a steady reduction in the levels of this drug. It can be assumed that the antielectroshock effect can be maintained by a gradual accumulation of the CBZ epoxide derivative, which has a longer half-life than the parent drug. To substantiate this hypothesis, it is necessary to measure the concentration of the CBZ-10,11-epoxide derivative in the plasma and brain of the tested animals. Regrettably, it was beyond our capabilities at that time. Further research is planned to test this hypothesis. Another possible explanation for the observed results is based on pharmacodynamic processes.

The primary mechanism of CBZ action is the use-dependent inhibition of voltage-gated sodium channels. CBZ has been shown to reduce the maximal amplitude of the sodium current, and the number of available channels, and to decelerate their recovery from the inactivation state [64]. CBZ exhibits a greater binding rate constant and lower affinity for the inactivated sodium channels when compared to phenytoin [65]. A transient reduction in sodium channel sensitivity to CBZ was demonstrated in the CA1 neurons of fully kindled rats. The inhibitory effect of CBZ on sodium channels recovered five weeks after the onset of kindling. This mechanism may contribute to the reduction in the anticonvulsant effect of CBZ observed in the 7 × 2 protocol. Nevertheless, the above in vitro phenomenon may not be indicative of CBZ ineffectiveness in vivo [66]. In animal experimental models of epilepsy and patients with drug-resistant epilepsy, the action of CBZ on the sodium channel function may be impaired [67]. It remains unclear whether chronic treatment with CBZ can influence the availability and action of sodium channels in healthy tissue.

Additional molecular mechanisms of CBZ action include the inhibition of calcium L channels, the modulation (primarily intensification) of potassium channel activity, the blockade of acetylcholine (Ach) receptors, and the increased release of serotonin. Although CBZ does not directly inhibit ionotropic glutamate receptors [68,69,70], it has been shown to inhibit veratridine-induced release of endogenous glutamate and to bind to the peripheral benzodiazepine receptor [52,65,70]. Interactions between CBZ and dopaminergic, serotoninergic, and GABAergic receptors have also been documented. However, it remains unclear whether this plays a role in the therapeutic effects of CBZ [53]. The epoxy metabolite of CBZ, epoxycarbamazepine, exhibits a comparable profile of activity [71].

The available pharmacodynamics data on chronic treatment with CBZ are limited and pertain only to a selected number of receptors and neurotransmitters. As expected, prolonged exposure to CBZ, which acts as an antagonist of an adenosine A1 receptor and an agonist of an adenosine A2 receptor, resulted in upregulation of the former and functional downregulation of the latter in cultured astrocytes and mouse brain [72,73]. Administration of CBZ at a dose of 25 mg/kg, both acutely and chronically (for a period of 3 weeks), resulted in an increase in intracellular Ach concentrations in the striatum and hippocampus of rats. A supra-effective dose of CBZ (100 mg/kg) was observed to inhibit the synthesis and release of Ach. As suggested by the authors of the article, the modest stimulation of ACh function may contribute, at least in part, to the antiepileptic and mood-stabilizing effects of CBZ [74]. A two-week exposure to CBZ resulted in a more than twofold reduction in ATP- or K+-stimulated glutamate release in astroglial cell culture [75]. An increase in the concentration of dopamine and its precursors, as well as its metabolites, has been observed in the striatum and hippocampus of rodents chronically treated with CBZ [76]. Subsequent studies demonstrated an increased concentration of kynurenic acid in the rat brain cortex and plasma, accompanied by a lack of change in the expression of kynurenic acid aminotransferase II, following prolonged administration of CBZ [77]. These preliminary results require further detailed verification in the context of the anticonvulsant action of CBZ.

To summarize, chronic treatment with CBZ did not affect its anticonvulsant efficacy in the majority of protocols. The exception was the 7 × 2 protocol, where the ED50 of the anticonvulsant was significantly higher than that of the control. No significant neurotoxic effects were observed in the chimney test or passive avoidance task during chronic treatment. Plasma concentrations of ASD were significantly reduced in three of the chronic protocols, while brain concentrations in all four chronic protocols compared to control. The reduced efficacy of CBZ in the 7 × 2 protocol may be due, at least in part, to metabolic mechanisms. On the other hand, functional mechanisms appear to be involved in maintaining the anticonvulsant effects of chronic carbamazepine against its lower concentrations in the remaining protocols. The results of the work presented here suggest that the results of studies based on a single administration of carbamazepine can be extrapolated to clinical settings to a degree comparable to the results obtained in chronic studies. Therefore, clinical trial designs for carbamazepine can be based on experimental studies regardless of the duration of drug administration.

## 4. Materials and Methods

### 4.1. Animals

The present study employed adult male Swiss mice that had not been subjected to any experimental procedures prior to the commencement of the study. The animals were maintained under standardized laboratory conditions, including housing in appropriately sized cages, a natural light–dark cycle of 12 h per day, a temperature range of 20–24 °C, and a humidity range of 45–65%. Air exchange was maintained at a rate of 15 times per hour. Additionally, the animals were provided with free access to food (chow pellets) and tap water. Following a seven-day period of acclimatization to laboratory conditions (7-day acclimatization), animals with a weight range of 20–26 g were randomly assigned to experimental groups. Each tested group comprised 8 to 10 mice. All investigations were conducted between 9.00 a.m. and 2.00 p.m. All procedures conducted in the study were performed in accordance with the European Union Directive 2010/63/EU for animal experimentation as well as the ARRIVE guidelines and were approved by the Local Ethical Committee (license nos. 10/2008 and 35/2009).

### 4.2. Drug

Carbamazepine (CBZ, Sigma Aldrich, St. Louis, MO, USA) was suspended in an aqueous solution of Tween 80. New solutions were prepared each day. The antiepileptic drug was administered intraperitoneally (i.p.) in a volume of 0.01 mL/g body weight. The chronic treatment with CBZ was conducted in accordance with four protocols:

1. A single injection was administered every 24 h for 7 days (7 × 1 subchronic treatment).
2. Two injections were administered daily every 12 h for 7 days (7 × 2 subchronic treatment).
3. A single injection was administered every 24 h for 14 days (14 × 1 chronic treatment).
4. Two injections daily were administered every 12 h for 14 days (14 × 2 chronic treatment).

Additionally, four control groups were included (1 × 1 acute treatment), one for each chronic schedule. The control animals were administered a vehicle in accordance with the respective chronic protocol. The last injection, however, contained CBZ administered 30 min prior to the commencement of the seizure test. The time of CBZ administration before testing and the range of drug doses were determined empirically based on the greatest increase in the seizure threshold. The control groups and the corresponding tested groups were subjected to electroconvulsions at the same time.

### 4.3. Maximal Electroshock Seizure Test

The anticonvulsant effect of CBZ was estimated in the maximal electroshock (MES) test in mice and expressed as the median effective dose (ED_50_). The MES test is a well-established animal model of tonic–clonic seizures commonly utilized in the preclinical evaluation of anticonvulsant properties of the tested molecules [78]. The median effective dose (ED_50_) represents the ability of the drug to protect 50% of animals against maximal electroshock-induced tonic hindlimb extension. A dose–response curve was calculated based on the percentage of mice protected (protection in less than 50%, around 50%, and more than 50% of animals) according to the methodology proposed by Litchfield and Wilcoxon [78]. The range of CBZ doses employed in the calculation of the ED_50_ values was 8–22 mg/kg (in the majority of cases, 12–20 mg/kg). These doses have been established in numerous previous experiments conducted over the course of our laboratory practice. The ED_50s_ values obtained in the fourth protocol were designated as ED_50_1 (1 × 1; single treatment protocol) and ED_50_2 (14 × 2; chronic protocol). Given that the longest and most intensive administration of a drug may result in the greatest accumulation and expression of adverse effects, further investigations were conducted employing two doses, i.e., ED_50_1 (14.2 mg/kg) and ED_50_2 (16.8 mg). The specifications of the rodent shocker generator (Type 221, Hugo Sachs Elektronik, Freiburg, Germany) and the comprehensive methodology were previously outlined by Borowicz-Reutt and Banach [17].

### 4.4. Chimney Test

The impact of both acute and chronic treatments with CBZ on motor performance in mice was evaluated using the chimney test [79]. The administration of the ASD was conducted at ED_50_1 (14.2 mg/kg) and ED_50_2 (16.8 mg/kg) doses derived from the MES test. Subsequently, CBZ was administered either once or in the 14 × 2 regime at both doses. This allowed a potential dose–response relationship to be observed. The results are presented as a percentage of animals that were unable to complete the test. The detailed methodology was previously described by Borowicz-Reutt and Banach [17].

### 4.5. Step-Through Passive Avoidance Task

The step-through passive avoidance test is regarded as a measure of long-term memory that is based on the natural reflex of being in the dark [80]. The mice were administered a single dose and a chronic treatment regimen (14 × 2) with CBZ at ED_50s_ values (ED_50_1 and ED_50_2). Subsequently, the mice were placed in an illuminated box (10 × 13 × 15 cm) that was connected to a larger dark box (25 × 20 × 15 cm), which was equipped with an electric grid floor. The animal’s entrance into the dark compartment was met with an appropriate electric foot shock (0.6 mA for 2 s). Mice that did not enter the dark compartment within 60 s were excluded from further participation in the experiment. Twenty-four hours later, the pre-trained mice were placed once more into the illuminated box and observed for up to 180 s. The animals were considered to have successfully completed the task if they avoided the dark compartment for the full observation period. The control group, comprising mice that had been treated with a vehicle only, did not enter the dark compartment within the observation period. The time taken by the mice to enter the dark compartment was recorded, and the median latencies (retention times) with 25th and 75th percentiles were calculated.

The detailed methodology was previously described by Borowicz-Reutt and Banach [17].

### 4.6. Measurement of Plasma and Brain Concentrations of Carbamazepine

The animals were administered CBZ in accordance with four distinct chronic protocols. A single treatment protocol was utilized as a control group. The final dose of CBZ in the chronic regimen was administered 30 min prior to the respective procedure, at the same time as scheduled for the electroconvulsive test. The precise methodology was previously outlined by Borowicz-Reutt and Banach [17].

The plasma and brain concentrations of CBZ were determined by fluorescence polarization immunoassay, utilizing the TDx analyzer and reagents in accordance with the manufacturer’s instructions (Abbott, Abbott Park, IL, USA). The results were expressed in μg/mL and subsequently computed as means ± SD of at least eight determinations.

### 4.7. Statistics

The ED_50_ (the dose that protects 50% of mice against tonic convulsions) was determined for CBZ in each experimental protocol. The ED_50_ values along with their corresponding 95% confidence limits were estimated through computerized log-probit analysis, in accordance with the methodology proposed by Litchfield and Wilcoxon [78]. A one-way analysis of variance (ANOVA) was employed to perform multiple comparisons of the ED_50_ values (±SEM) from the MES test, followed by the post hoc Tukey test.

Qualitative variables derived from the chimney test were evaluated using Fisher’s exact probability test, while the results obtained from the step-through passive avoidance task were subjected to statistical analysis employing the Kruskal–Wallis nonparametric analysis of variance (ANOVA), followed by post hoc Dunn’s test.

One-way analysis of variance (ANOVA) was employed to evaluate plasma and brain concentrations of CBZ, with Dunnett’s post hoc test used to identify any significant differences. The level of significance was set at *p* ≤ 0.05, in accordance with the recommendations of Hamada [81].

## 5. Conclusions

Following prolonged administration of CBZ, a mere transient diminution in the anticonvulsant efficacy was observed in the MES test, despite a notable decline in the plasma and brain concentrations of the drug. Concomitantly, tolerance to neurotoxic effects on motor coordination was observed. It seems reasonable to suggest that the above results can be explained by pharmacokinetic mechanisms, including the possibility of CBZ metabolite accumulation. Nevertheless, the possibility of a pharmacodynamic phenomenon with regard to the toxicity of CBZ cannot be excluded.

The anticonvulsant and neurotoxic effects of carbamazepine were observed to be similar following both single and chronic administration. In light of the aforementioned findings, it is evident that conducting experimental chronic studies on carbamazepine is not a prerequisite for drawing inferences and extrapolating results to clinical conditions. Thus, previous and current animal studies conducted in a single-dose model can be considered reliable for the planning of clinical trials. From an experimental point of view, the lack of a need to run chronic studies with CBZ allows for a reduction in the size of animal groups, which aligns with ethical guidelines.

## Figures and Tables

**Table 1:** Effects of acute and chronic treatments with carbamazepine (CBZ) on motor performance in the chimney test.

Drug and Dose (mg/kg)	Treatment Protocol	Animals Impaired (%)
Vehicle	1 × 1	0
Vehicle	14 × 2	0
CBZ 14.2 (ED501)	1 × 1	0
CBZ 14.2	14 × 2	0
CBZ 16.8 (ED502)	1 × 1	0
CBZ 16.8	14 × 2	0

Data are expressed as the percentages of animals that failed to perform the chimney test. Statistical analysis of the data was performed using Fisher’s exact probability test. CBZ—carbamazepine; treatment protocols: 1 × 1, 1 injection; 14 × 2, 2 injections daily for 14 days; ED_50_–median effective dose.

**Table 2:** Effects of acute and chronic treatments with carbamazepine (CBZ) on long-term memory in mice.

Drug and Dose (mg/kg)	Treatment Protocol	Retention Time (s)
Vehicle	1 × 1	180 (180; 180)
Vehicle	14 × 2	180 (180; 180)
CBZ 14.2 (ED501)	1 × 1	180 (162; 180)
CBZ 14.2	14 × 2	180 (180; 180)
CBZ 16.8 (ED502)	1 × 1	180 (180; 180)
CBZ 16.8	14 × 2	180 (115; 180)

Data are expressed as median retention time (with 25th and 75th percentiles), during which the animals avoided the dark compartment in the step-through passive avoidance task. Statistical analysis of the data was performed using the nonparametric Kruskal–Wallis ANOVA test followed by Dunn’s post hoc test. CBZ—carbamazepine; treatment protocols: 1 × 1, 1 injection; 14 × 2, 2 injections daily for 14 days; ED_50_–median effective dose.

## Data Availability

The data presented in this study are available in the authors’ own database.
